# The influence of childhood trauma and chronotype on suicide attempts in Chinese emerging adults with severe depressive symptoms

**DOI:** 10.1186/s40359-023-01472-0

**Published:** 2024-01-03

**Authors:** Yi Yu, Yi Feng, Shicun Xu, Amanda Wilson, Chang Chen, Xi Ling, Runsen Chen, Yuanyuan Wang

**Affiliations:** 1 School of Psychology, Center for Studies of Psychological Application, and Guangdong Key Laboratory of Mental Health and Cognitive Science, South China Normal University, Guangzhou, China; 2 Mental Health Center, Central University of Finance and Economics, Beijing, China; 3 Faculty of Psychology, Beijing Normal University, Beijing, China; 4 Northeast Asian Research Center, Jilin University, Changchun, China; 5 Department of Population, Resources and Environment, Northeast Asian Studies College, Jilin University, Changchun, China; 6 China Center for Aging Studies and Social-Economic Development, Jilin University, Changchun, China; 7 Division of Psychology, Faculty of Health and Life Sciences, De Montfort University, Leicester, UK; 8 Vanke School of Public Health, Tsinghua University, Beijing, China

**Keywords:** Childhood trauma, Chronotype, Suicide attempts, Severe depressive symptoms

## Abstract

**Background:**

Studies have investigated how adults with severe depressive symptoms are more likely to attempt suicide, and these adults often have traumatic experiences and chaotic sleep/wake rhythms. Thus, this study using Latent class analysis aimed to investigate the relationship between childhood trauma class, chronotype, and suicide attempts among emerging adults with severe depressive symptoms.

**Methods:**

This study was conducted among emerging adults with severe depressive symptoms covering 63 Universities in Jilin Province, China. A total of 1,225 emerging adults (mean age = 19.6 ± 1.78) constructed the final sample. In addition to measuring socio-demographic characteristics, the Childhood Trauma Questionnaire-Short Form, the Single-Item Chronotyping, and a single item for suicide attempts were used to evaluate childhood trauma, chronotype, and suicide attempts, respectively. Latent class analysis was applied to identify the classes of childhood trauma within emerging adults who had severe depressive symptoms. Hierarchical logistic regression models were run to investigate the effects of socio-demographic characteristics, chronotype, and childhood trauma class on suicide attempts.

**Results:**

Three latent classes were identified: the Low-risk for childhood trauma class, the Neglect class, and the High-risk for childhood abuse class. Those who suffered sexual, emotional, and physical abuse at the same time were divided into the High-risk for childhood abuse class, and were significantly more likely to experience suicide attempts than those in the Neglect class (*OR* = 1.97, 95%*CI* = 1.34–2.89, *p* < 0.001) and the Low-risk for childhood trauma class (*OR* = 2.28, 95% *CI* = 1.50-3.46, *p* < 0.001). In terms of chronotype, the results showed that the chaotic type was a risk factor for suicide attempts when compared with the evening type (OR = 0.46, 95%*CI* = 0.27–0.78, *p* < 0.01), the moderately active type (OR = 0.53, 95%*CI* = 0.31–0.89, *p* < 0.05), and the daytime type (OR = 0.42, 95%*CI* = 0.21–0.86, *p* < 0.05). Overall, the significant risk factors for suicide attempts included being female, living in an urban area, having experienced sexual, emotional, and physical abuse simultaneously, and having a chaotic chronotype.

**Conclusion:**

Emerging adults suffering sexual, emotional, and physical abuse at the same time and identifying with chaotic chronotype showed a higher risk of attempting suicide. The findings provided a clinical reference to quickly identify those at high risk of suicide attempts among emerging adults with severe depressive symptoms.

## Introduction

Depression is the second leading cause of chronic disability globally, and has been identified as an increasingly concerning public health problem [1]. According to the World Health Organization (WHO), the rate of depression increased by over 25% during the first year of the COVID-19 pandemic (World Health Organization [WHO], 2022). This is also true in emerging adults, where depression rates have risen over the past few years, which has resulted in a social concern for this group because of the importance of the developmental period for cognitive and emotional processing [2]. A meta-analysis including studies from multiple countries suggested that about 34% of emerging adults suffered from depressive symptoms [3], and approximately 6.6% of emerging adults in China suffered from severe depressive symptoms [4], compared with 8% in Turkey and 13% in Australia [5]. Worryingly, suicide rates are higher among emerging adults with severe depressive symptoms [6]. According to a mental health surveillance report, about 8% of emerging adults with severe depressive symptoms died by suicide [7]. Suicide attempt, referring to a form of self-harm with the intention of dying, is the most powerful predictor of death by suicide (Hill et al., 2020), and is common among adults with severe depressive symptoms [8]. A US study reported that 22.5% of emerging adults suffering from depression had attempted suicide in 6 months before being hospitalized for mental health problems [9]. Another study in China showed that 19.5% of those with severe depressive symptoms had previously attempted suicide [10]. Therefore, it is crucial to pay attention to suicide attempts among emerging adults with severe depressive symptoms and identify specific risk factors to prevent death by suicide.

Many studies have suggested that being female [11], having a younger age [12], living in an urban residence [13], having poor family cohesion, having a poor relationship with parents [14], and experiencing insomnia [12] are all common risk factors for suicide attempts. Furthermore, childhood trauma and chronotype are also considered important predictors of suicide attempts for those with severe depressive symptoms [15, 16].

Furthermore, a previous study showed that emerging adults with severe depressive symptoms were more likely to have a history of adverse childhood experiences [17, 18], such as childhood trauma [19], which can contribute to an increased risk of suicide attempts [20]. Childhood trauma is an umbrella term that encompasses five dimensions: emotional abuse, emotional neglect, physical abuse, physical neglect, and sexual abuse [21]. Different dimensions of childhood trauma exert different effects on suicide attempts in those with severe depressive symptoms. For example, Medeiros and colleagues [22] suggested that all five childhood traumatic experiences predict suicide attempts. However, sexual abuse and physical neglect directly affect suicide attempts, and the associations between other dimensions of childhood trauma and suicide attempts are mediated through psychache (unbearable psychological pain) and dissociation [23]. Sarchiapone and colleagues [24] found that only emotional neglect was a significant predictor of suicide attempts. Among emerging adults with severe depressive symptoms, the existing research has shown that sexual abuse is linked to suicide attempts [25], while the associations between other dimensions of childhood trauma and suicide attempt need to be further explored.

Suicide attempts have been further associated with chronotype. Chronotype is defined as a personal preference and biological tendency for rest and activity time [5, 26], mainly including morning type, evening type, highly active type, moderately active type, daytime type, and daytime sleepy type [27]. Most of the studies on the subject usually focused on the different effects on suicidality between morning type and evening type [28, 29]. Compared with the morning type, the evening type has been found to be more strongly associated with suicidal ideation and suicide attempts in individuals with severe depressive symptoms [16, 30]. To date, most research in the field of chronotype has well proved that the disturbance of circadian rhythms is common in emerging adults with severe depressive symptoms [31]. However, research has been limited to comparisons between morning and evening types, with little reported on the associations between other types of chronotype, especially the chaotic chronotype.

It is possible among emerging adults that all three factors behind suicide attempts, severe depressive symptoms, childhood trauma, and chronotype, are related. For example, a study found that participants who experienced more adverse events in childhood (including childhood trauma) were more likely to be later chronotypes [32]. Thus, exploring the relationship between childhood trauma and chronotype, as well as their interaction with suicide attempts, is necessary to clarify the effects of childhood trauma and chronotype on the likelihood of suicide attempts.

The above-reviewed studies used a variable-centered method, focusing on the specific dimension of childhood trauma and its influence on suicide attempts [33]. An variable-centered approach can lead to an overestimation of the effects of single dimensions and overlook the impact of the co-occurrence and overlap of the dimensions of childhood trauma [34]. Therefore, it is crucial to pay attention to the heterogeneity of individuals within different childhood trauma dimensions and to identify the different at-risk groups for suicide attempts [35]. Latent class analysis (LCA) is a statistical method with categorical outcomes, that is used to identify latent classes within a population. Classes can be regarded as hidden subgroups that represent the heterogeneity of a population. Individuals with similar characteristics are grouped together [36]. Therefore, LCA is a person-centered method [37], in contrast to the dominant variable-centered tradition, which assumes homogeneity in the population and explores the impact of individual independent variables on the outcome variables [38]. While LCA is widely applied in the field of childhood trauma [34, 39], there are few studies exploring the relationship between childhood trauma class and suicide attempts using this method. For example, Luk and colleagues [40] suggested that the multiple and persistent abuse class, which is characterized by high probabilities of all abuse, is associated with an increased risk of suicide attempts. Thus, by utilizing LCA to identify childhood trauma classes, the researchers can make practical suggestions to better screen for suicide attempts in Chinese emerging adults with severe depressive symptoms, which cannot be achieved using a variable-centered approach alone.

This study, therefore, aimed to explore the relationship between childhood trauma and chronotype on suicide attempts to clarify the effects of childhood trauma and chronotype on suicide attempts. The first aim of this study was to find the risk factors for suicide attempts among emerging adults with severe depressive symptoms by identifying the different risk ratings of suicide attempts for their classes of childhood trauma. The second aim was to systematically investigate the effects of several chronotypes on suicide attempts, especially the chaotic type.

## Method

### Participants

The data was derived from a large cross-sectional study of 96,218 students from 63 Universities in Jilin Province, China, using a convenience sampling method, which placed an advertisement for participants from the Jilin Provincial Department of Education. Those interested in the advertisement used the Quick Response Code (QR Code) and were able to access the questionnaires only after providing online consent to participate. Students who were 15 years old or older and diagnosed with severe depressive symptoms (the score of the Patient Health Questionnaire-9 ≥ 20 [41] were recruited. 1,225 participants who met the inclusion criteria were selected and divided into two groups, including the suicide attempts group and the non-suicide attempts group. 98 participants had missing data on the socio-demographic variables, which were excluded from the statistical analysis. And this study was conducted in accordance with the 1964 Helsinki Declaration and approved by the Ethical Committee of Jilin University.

### Measure

#### Socio-demographic characteristics

The socio-demographic characteristics included age, sex, residence, relationship with father/mother, and family harmony. Sex was assessed in binary form as “Male” or “Female”. Residence was also dichotomously assessed as “Urban” or “Rural”. Age, relationship with parents, and family harmony were all continuous variables. Relationship with father/mother was measured by a single item with 5 options. A lower score represented a better relationship with parents, with scores ranging from 1 (“very good”) to 5 (“very bad”) [14]. In addition, participants were asked about their family harmony using an item ranging from 1 (“very bad”) to 10 (“very good”) [42].

#### Depression

The Patient Health Questionnaire-9 (PHQ-9), is a 9-item self-report scale. It is one of the most commonly used scales to assess depressive symptoms [41]. The Cronbach’s alphas in the Chinese version were 0.86 [43], and 0.89 in this study (though note the reliability may have been impacted by recruiting those with a score equal to or higher than 20). According to the criteria of the Diagnostic and Statistical Manual of Mental Disorders (DSM-IV), the PHQ-9 screens for depression in emerging adults. Each item is presented with a 4-point Likert scale, ranging from 0 (never) to 3 (almost every day), to assess the frequency of symptoms in the last two weeks. A higher total score represents a higher level of depressive symptoms. The score is 5–9 for mild depression, 10–14 for moderate depression, 15–19 for moderately severe depression, and ≥ 20 for severe depression. In this study, participants who scored ≥ 20 points were classified as meeting the criteria for severe depressive symptoms.

#### Childhood trauma

The Childhood Trauma Questionnaire-Short Form (CTQ-SF), is a 28-item Likert scale, and was used in this study to screen emerging adults for traumatic experiences in childhood [44]. The CTQ-SF consists of five subscales, including sexual abuse, emotional abuse, physical abuse, physical neglect, and emotional neglect. Each subscale has five items ranging from 1 (“never true”) to 5 (“very often true”). Different optimal cut-off scores are recommended to screen positive participants in each subscale. The cut-off scores are ≥ 13 in the emotional abuse subscale, ≥ 10 in the physical abuse subscale, ≥ 8 in the sexual abuse subscale, ≥ 10 in the physical neglect subscale, and ≥ 15 in the emotional neglect subscale. The Cronbach’s alpha of the Chinese version in previous study was 0.79 [45]. In this study, Cronbach’s alpha of this scale was 0.63–0.88 for the five subscales and 0.90 for the composite childhood abuse scores.

#### Chronotype

Chronotype was measured as a categorical variable using the Single-Item Chronotyping (SIC), in which participants select one item with seven different options to choose from [27], though as a single measure, it is hard to assess its reliability. Emerging adults were asked to choose the appropriate option based on their preference of timing for rest and activity. The seven options included: morning type (decreased alertness throughout the day), evening type (increased alertness throughout the day), highly active type (persistent high alertness), moderately active type (persistent low alertness), daytime type (an alert peak in the afternoon), daytime sleepy type (decreased alertness in the afternoon), and the other type (unspecified). The other type refers to a preference timing that is too unstable to be classified into one of the other six types. Therefore, it is called the chaotic type.

#### Suicide attempts

Suicide attempts were measured by the Chinese version of the Suicidal Behaviors Questionnaire-Revised (SBQ-R), which is a 4-item self-report scale with high reliability (0.73) [46]. Based on the original version, only Item 1 (i.e., “Have you ever thought about or attempted to kill yourself”) was used in this study to assess whether participants had attempted suicide in the past [47]. Specifically, participants with a score of 1–3 (“never”- “I have had a plan”) in this item were assigned to the non-suicide attempts group, while those with a score of 4 (“I had attempted suicide”) were assigned to the suicide attempts group. Item 1 of the SBQ-R has high sensitivity (100%) and specificity (96%) [47]. In the current study, Cronbach’s alpha was 0.80.

To test the common method bias, the Harman single factor test was performed. The results showed that the first latent factor accounted for 22.79% of assigned classes, indicating that the common method bias was unlikely an issue in our study.

### Statistical analysis

First, the t-test and chi-squared test were used to explore the differences between the suicide attempts group and the non-suicide attempts group in terms of socio-demographic characteristics and chronotype. Next, the LCA was used to classify participants into mutually exclusive groups according to their co-occurrence of childhood trauma. After fitting several models with an increased number of classes, the model fitting parameters of each model were compared to select the optimal one [48]. The model fitting parameters of the LCA included Akaike Information Criteria (AIC), Bayesian Information Criterion (BIC), adjusted BIC (aBIC), Entropy, Bootstrap Likelihood Ratio (BLRT), and Lo-Mendell Ruben test (LMR). Specifically, lower values of AIC, BIC, and aBIC commonly indicated a better fit. An entropy above 0.6 was considered acceptable [37]. Moreover, LMR and BLRT were used to examine whether the model with k classes was significantly different from the model with k-1 classes [49]. Based on the above fitting indexes, the proportion of the smallest class (larger than 5%), and the substantial meaningfulness, this study determined the optimal model and named each class based on its unique characteristics. Next, a chi-squared test was run between the suicide attempts group and the non-suicide attempts group in terms of childhood trauma classes. Then, in order to test the relationship between childhood trauma class and chronotype, the Lambda correlation analysis and the chi-squared test were also conducted. Last, two 3-step hierarchical logistic regression analysis were performed to evaluate the effects of socio-demographic characteristics, chronotype, and childhood trauma class on suicide attempts for adults with severe depressive symptoms. All statistical tests were two-tailed tests with a significance level of *p* < 0.05. Statistics were conducted using SPSS 25.0, Mplus 7.0, and R software 4.0.2.

## Result

### Socio-demographic characteristics

As shown in Table 1, there were 1,225 emerging adults self-reporting severe depressive symptoms in the final sample. Among the 1,225 participants, the average age was 19.6 years old (*SD* = 1.78). 639 (52.2%) were female, 560 (45.7%) lived in rural areas, and 310 (25.3%) participants self-reported suicide attempts. In terms of the chronotype, most of the participants tended to self-report being the evening type (33.9%) and moderately active type (31.3%).

**Table 1:** Socio-demographic characteristics of the participants

Variables	Total	Non-suicide attempts	Suicide attempts	*t*/*χ*^*2*^
	(*n* = 1,225)	(*n* = 915)	(*n* = 310)	
Age, mean, (SD)	19.6 (1.78)	19.7 (1.81)	19.6 (1.69)	**0.94** ^***^
Sex				
Male	586 (47.8%)	467 (51.0%)	119 (38.4%)	**14.85** ^***^
Female	639 (52.2%)	448 (49.0%)	191 (61.6%)	
Residence				
Urban	665 (54.3%)	482 (52.7%)	183 (59.0%)	3.77
Rural	560 (45.7%)	433 (47.3%)	127 (41.0%)	
Family harmony, mean, (SD)	6.04 (2.93)	6.32 (2.82)	5.24 (3.12)	**5.41** ^***^
Relationship with father, mean, (SD)	2.27 (1.11)	2.19 (1.08)	2.50 (1.18)	**-4.53** ^***^
Missing	60 (4.9%)	34 (3.7%)	26 (8.4%)	
Relationship with mother, mean, (SD)	1.87 (0.97)	1.78 (0.91)	2.13 (1.12)	**-5.97** ^***^
Missing	38 (3.1%)	19 (2.1%)	19 (6.1%)	
Chronotype				
Morning type	52 (4.2%)	38 (4.2%)	14 (4.5%)	**16.06** ^*^
Evening type	415 (33.9%)	323 (35.3%)	92 (29.7%)	
Highly active type	66 (5.4%)	46 (5.0%)	20 (6.5%)	
Moderately active type	383 (31.3%)	286 (31.3%)	97 (31.3%)	
Daytime type	104 (8.5%)	84 (9.2%)	20 (6.5%)	
Daytime sleepy type	107 (8.7%)	79 (8.6%)	28 (9.0%)	
Chaotic type	98 (8.0%)	59 (6.4%)	39 (12.6%)	

*Note.* * *p* < 0.05.** *p* < 0.01.*** *p* < 0.001

### The classes of childhood trauma

According to the comparison of fitting indexes (see Table 2), the three-class model was the most optimal because it had the lowest BIC and aBIC values. The BLRT and LMR statistics showed that the three-class model was a better fit than the two-class model, while there was no significant difference between the three-class and the four-class model. Furthermore, the proportion for the lowest classes in the four-class model was only 4.1%, which was less than the optimal value of 5% [50]. Although the entropy value (0.69) of the three-class model was lower than the cut-off standard (0.8), it was still an acceptable standard (0.6) [51].

**Table 2:** Model fitting parameters of Latent class analysis on childhood trauma

Model	Log-likelihood	AIC	BIC	aBIC	BLRT(*p*)	LMR(*p*)	Entropy	Sample proportion (%) per class
2	-3143.78	6309.57	6365.79	6330.85	< 0.001	< 0.001	0.70	55.6/44.4
3	**-3100.11**	**6234.22**	**6321.10**	**6267.10**	**< 0.001**	**< 0.001**	**0.69**	**49.8/17.6/32.6**
4	-3093.69	6233.38	6350.07	6277.86	0.216	0.061	0.74	4.1/48.7/32.7/14.5
5	-3086.82	6231.64	6379.85	6287.73	0.050	0.265	0.74	6.5/13.5/49.3/6.5/2.4

*Note.* AIC = Akaike information criterion. BIC = Bayesian information criterion (BIC). aBIC = Sample-adjusted BIC. LMR = Lo-Mendell Ruben test. BLRT = Bootstrap Likelihood Ratio test. Selected category was shown in bold

**Fig. 1:**
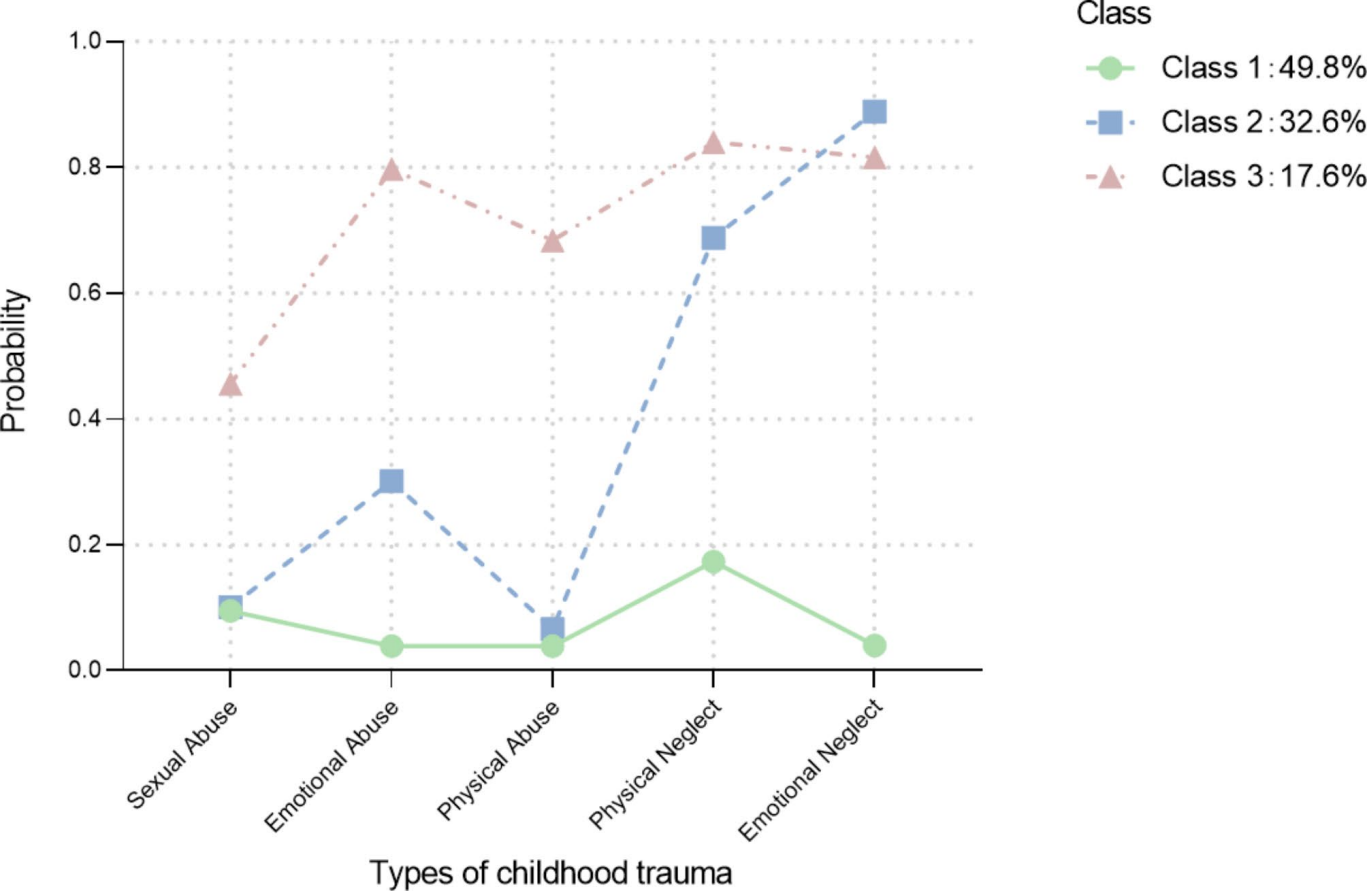
The probability of latent classes of childhood trauma *Note.* Class 1 = Low-risk for childhood trauma. Class 2 = Neglect. Class 3 = High-risk for childhood abuse

The three classes of childhood trauma are shown in Fig. 1. Specifically, Class 1 was characterized by the lowest probability of all abuse and neglect (0.04–0.17) and defined as “Low-risk for childhood trauma”, covering 49.8% of the participants. Class 2 was defined as “Neglect”, which was characterized by high probabilities of physical neglect (0.69) and emotional neglect (0.89). However, the probability of abuse was low (0.07–0.30), covering 32.6% of the participants. Class 3 was defined as “High-risk for childhood abuse”, which referred to the highest probability of all three dimensions of abuse, with physical abuse (0.68), emotional abuse (0.80), and sexual abuse (0.46) covering 17.6% of the participants. This class also demonstrated a higher probability of neglect (0.82 and 0.84) than Class 1 “Low-risk for childhood trauma”.

### Comparisons of the suicide attempt and the non-suicide attempts groups

**Fig. 2:**
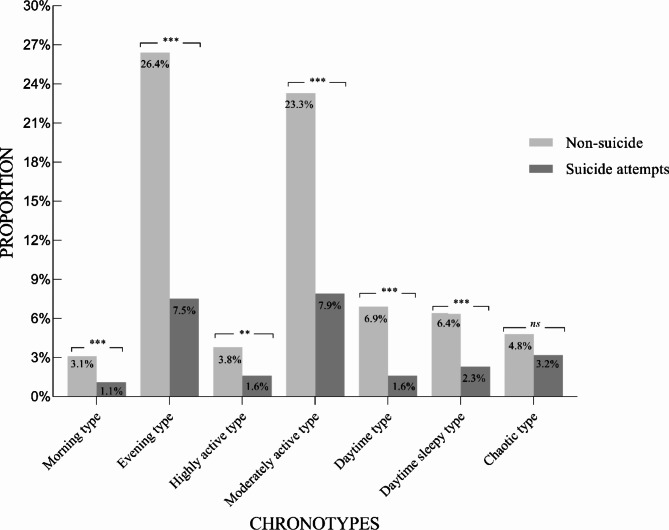
Differences of chronotype between the Non-suicide attempts and the Suicide attempts groups *Note.* * *p* < 0.05.** *p* < 0.01.*** *p* < 0.001. *ns* = non-significant

**Fig. 3:**
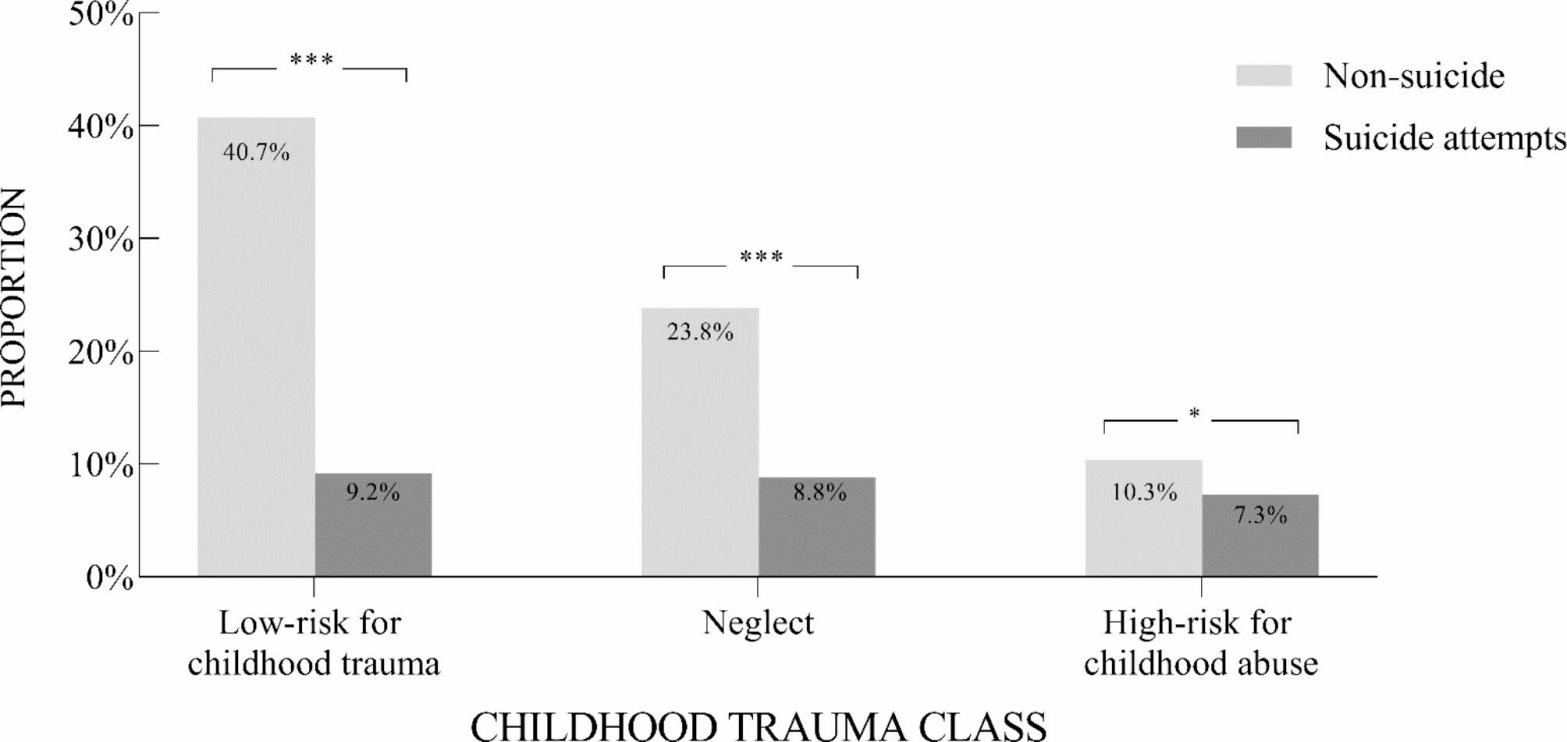
Differences of childhood trauma classes between the Non-suicide attempts and the Suicide attempts groups *Note.* Class 1 = Low-risk for childhood trauma. Class 2 = Neglect. Class 3 = High-risk for childhood abuse * *p* < 0.05. ** *p* < 0.01. *** *p* < 0.001

As seen in Fig. 2, in terms of chronotype, there were significant differences between the suicide attempts and the non-suicide attempts groups in the morning type (*χ*^*2*^ = 38, *p* = 0.001), evening type (*χ*^*2*^ = 323, *p* < 0.001), highly active type (*χ*^*2*^ = 46, *p* < 0.01), moderately active type (*χ*^*2*^ = 286, *p* < 0.001), daytime type (*χ*^*2*^ = 84, *p* < 0.001), and daytime sleepy type (*χ*^*2*^ = 79, *p* < 0.001). No significant differences were found in the chaotic type (*χ*^*2*^ = 59, *p* = 0.055). Figure 3 illustrates the different proportions of childhood trauma classes between the suicide attempts and the non-suicide attempts groups. In the suicide attempts group, the proportion of all three classes was significantly lower than the non-suicide attempts group (the High-risk for childhood abuse class: *χ*^*2*^ = 89, *p* < 0.05; the Neglect class: *χ*^*2*^ = 291, *p* < 0.001; the Low-risk for childhood trauma class: *χ*^*2*^ = 498, *p* < 0.001). Additional statistical analysis on the association between chronotype and childhood trauma class suggested that there was no significant correlation (*r* = 0.01, *p* = 0.49; *χ*^*2*^ = 13.57, *p* = 0.33). That is, the impact on suicide attempts was independent.

### Risk factors related to suicidal attempts

Logistic regression models were built to investigate the effects of main variables on suicide attempts (see Table 3). Model 1 shows that urban residence (*OR* = 1.41, 95%*CI* = 1.06–1.87, *p* < 0.05) and poor relationship with mother (*OR* = 1.22, 95%*CI* = 1.03–1.44, *p* < 0.05) were positively related to suicide attempts, while being male (*OR* = 0.55, 95%*CI* = 0.41–0.73, *p* < 0.001) and having good family harmony (*OR* = 0.92, 95%*CI* = 0.86–0.98, *p* < 0.01) were protective factors. Model 2 shows that in addition to the above factors, when compared with the chaotic type, the evening type (*OR* = 0.47, 95%*CI* = 0.28–0.79, *p* < 0.01), the moderately active type (*OR* = 0.53, 95%*CI* = 0.32–0.90, *p* < 0.05) and the daytime type (*OR* = 0.44, 95%*CI* = 0.22–0.89, *p* < 0.05) were also significantly related to a decrease in the probability of suicide attempts. As shown in Model 3, participants in the High-risk for childhood abuse class were significantly more likely to experience suicide attempts than those in the Neglect class (*OR* = 1.97, 95%*CI* = 1.34–2.89, *p* < 0.001) and the Low-risk for childhood trauma class (*OR* = 2.28, 95%*CI* = 1.50–3.46, *p* < 0.001). Compared with the evening type (*OR* = 0.46, 95%CI = 0.27–0.78, *p* < 0.01), the moderately active type (*OR* = 0.53, 95%*CI* = 0.31–0.89, *p* < 0.05), and the daytime type (*OR* = 0.42, 95%*CI* = 0.21–0.86, *p* < 0.05), the chaotic type was a risk factor for suicide attempts. There was no significant impact of the other types of chronotype on suicide attempts, and this did not change depending on the chronotype used for comparison. In addition, the impact of being male (*OR* = 0.54, 95%*CI* = 0.40–0.73, *p* < 0.001) and living in an urban residence (*OR* = 1.36, 95%*CI* = 1.02–1.82, *p* < 0.05) on suicide attempts showed significance in this final model. After the addition of childhood trauma class, however, the impact of the relationship with the mother (*OR* = 1.15, 95%*CI* = 0.96–1.37, *p* = 0.124) and family harmony (*OR* = 0.94, 95%*CI* = 0.88-1.00, *p* = 0.055) was no longer significant.

**Table 3:** Hierarchical logistic regression on chronotype, childhood trauma class in predicting suicide attempts

	Model 1		Model 2		Model 3 − 1		Model 3 − 2	
	*OR*	95%*CI*	*OR*	95%*CI*	*OR*	95%*CI*	*OR*	95%*CI*
Age	0.96	0.89–1.05	0.96	0.88–1.04	0.95	0.88–1.04	0.95	0.88–1.04
Male (Ref = Female)	**0.55** ^***^	0.41–0.73	**0.54** ^***^	0.40–0.72	**0.54** ^***^	0.40–0.73	**0.54** ^***^	0.40–0.73
Urban (Ref = Rural)	**1.41** ^*^	1.06–1.87	**1.42** ^*^	1.06–1.89	**1.36** ^*^	1.02–1.82	**1.36** ^*^	1.02–1.82
Better family harmony	**0.92** ^**^	0.86–0.98	**0.92** ^**^	0.86–0.98	0.94	0.88-1.00	0.94	0.88-1.00
Worse relationship with father	1.02	0.87–1.21	1.02	0.86–1.20	0.99	0.84–1.17	0.99	0.84–1.17
Worse relationship with mother	**1.22** ^*^	1.03–1.44	**1.22** ^*^	1.03–1.44	1.15	0.96–1.37	1.15	0.96–1.37
Chronotype (Ref = Chaotic type)								
Morning type			0.65	0.28–1.48	0.64	0.28–1.48	0.64	0.28–1.48
Evening type			**0.47** ^**^	0.28–0.79	**0.46** ^**^	0.27–0.78	**0.46** ^**^	0.27–0.78
Highly active type			0.76	0.36–1.60	0.7	0.33–1.48	0.7	0.33–1.48
Moderately active type			**0.53** ^*^	0.32–0.90	**0.53** ^*^	0.31–0.89	**0.53** ^*^	0.31–0.89
Daytime type			**0.44** ^*^	0.22–0.89	**0.42** ^*^	0.21–0.86	**0.42** ^*^	0.21–0.86
Daytime sleepy type			0.7	0.37–1.35	0.68	0.35–1.31	0.68	0.35–1.31
CT class (Ref = Class 1)								
Class 2					1.16	0.80–1.67		
Class 3					**2.28** ^***^	1.50–3.46		
CT class (Ref = Class 2)								
Class 1							0.86	0.60–1.25
Class 3							**1.97** ^***^	1.34–2.89
*R* ^*2*^_*CS*_	0.05		0.059		0.073		0.073	
*R* ^*2*^_*N*_	0.075		0.089		0.109		0.109	

*Note.* Class 1 = Low-risk for childhood trauma. Class 2 = Neglect. Class 3 = High-risk for childhood abuse. *OR* = Odds ratio. *CI* = Confidence interval. *R*^*2*^_*CS*_ = Cox & Snell R Square. *R*^*2*^_*N*_ = Nagelkerke R Square

* *p* < 0.05. ** *p* < 0.01. *** *p* < 0.001

## Discussion

This is a large cross-sectional study that performed an exploration of the co-occurrence and overlap of childhood trauma utilizing LCA, and identified three latent classes based on the co-occurrence of childhood trauma dimensions, namely the High-risk for childhood abuse class, the Neglect class, and the Low-risk for childhood trauma class. The High-risk for childhood abuse class could predict suicide attempts after controlling for other variables. This study also examined the effects of chronotype on suicide attempts. Compared with the evening type, moderately active type, and daytime type, the chaotic type was a risk factor for suicide attempts. Furthermore, being female and living in an urban residence usually were significantly associated with suicide attempts.

Given the heterogeneity of a population, LCA is a person-centered method, grouping similar people together based on the set of participants’ responses to indicators. In this study, LCA was applied to classify participants into three distinct classes: the High-risk for childhood abuse class, the Neglect class, and the Low-risk for childhood trauma class. Similar to the present results, Chen and colleagues [52] also found that the 3-class model was the optimal model and defined each class as “Low abuse and neglect”, “High neglect”, and “High abuse and neglect”. The specific probabilities of the five dimensions in each class were different in our study. In the High-risk for childhood abuse class, participants showed the highest probability of all three dimensions of abuse with sexual abuse, emotional abuse, and physical abuse, which indicated a high co-occurrence of these three abuse types. This finding confirmed the strong association and co-occurrence between physical abuse and emotional abuse, as well as physical abuse and sexual abuse in previous studies [53, 54]. A possible explanation may be that emerging adults who experience physical abuse often come from unharmonious families, with issues such as parental violence and divorce, crime, substance abuse and psychiatric disorders, all of which can also exacerbate the emotional and sexual abuse [33, 55]. About 216 participants (17.6%) were included in the High-risk for childhood abuse class. In the Neglect class, participants showed the highest probability of neglect, especially emotional neglect. Furthermore, in this class, all forms of abuse were lower than the High-risk for childhood abuse class, but higher than the Low-risk for childhood trauma class. 399 emerging adults (32.6%) were divided into the Neglect class, suggesting that physical and emotional neglect are the most common dimensions of childhood trauma [56, 57], and usually occur simultaneously. A community sample showed that individuals with emotional neglect were 12.2 times more likely to suffer physical neglect than those without emotional neglect [58].

Emerging adults with severe depressive symptoms, who were classified into the High-risk for childhood abuse class, were more likely to attempt suicide. As far as the co-occurrence and overlap of childhood trauma are concerned, compared with the co-occurrence of physical neglect and emotional neglect, the co-occurrence of emotional abuse, physical neglect, and physical abuse showed the strongest negative impact on life enjoyment and hope for the future, leading to suicide attempts [59]. In addition, abuse can be argued to be more dangerous than neglect as it disrupts the brain’s normal neurological, hormonal, inflammatory, and immune physiology. For example, a previous study has shown that victims of physical, emotional, and sexual abuse exhibited sensitive autonomic responses, increased inflammation and pituitary-adrenal responses, that, in combination with other psychiatric problems, such as severe depressive symptoms, result in suicide attempts [60, 61]. Preliminary research also suggested that those without PTSD may have a reduced serum level of high-density lipoprotein levels, on the A allele of tropomyosin-related kinase receptor B (which impacts both learning and memory) when compared to those with PTSD [62]. Considering the number of childhood trauma, the cumulative effects of abuse and neglect can also lead to increased suicide attempts [63, 64]. However, contrary to most studies [22, 65], emerging adults in the Neglect class were not inclined to attempt suicide. This discrepancy could be attributed to a lack of control for other experiences of abuse in the previous study. In this study, our results demonstrated the unstable impact of physical neglect and emotional neglect on suicide attempts. A cohort study has suggested similar findings that severe physical neglect and emotional neglect are not significantly correlated to an increased risk of suicide attempts [66]. Relative to those in the other two classes, emerging adults in the Low-risk for childhood trauma class were the least likely to attempt suicide, which is similar to prior person-centered studies [40, 67, 68]. Thus, paying attention to emerging adults’ multiple childhood trauma experiences, particularly sexual, emotional, and physical abuse, is essential to develop effective interventions to reduce the risk of suicide attempts.

Based on the sleep-wake cycle, the hormones melatonin, body temperature, and cortisol, the chronotype is usually classified into three dimensions consisting of the morning type, evening type, and neither type [69]. Most of the previous research has only examined the different effects of these three chronotypes on suicide attempts using unidimensional questionnaires [26, 70]. However, the disturbance of circadian rhythms has been well proved in emerging adults with severe depressive symptoms [31], which leads to the fact that most depressed adults (about 60%) cannot be classified into morning type and evening type alone [71]. Therefore, this study expanded chronotype into seven types and found that compared with the evening type, the moderately active type, and the daytime type, the chaotic type was a risk factor for suicide attempts. Contrary to previous findings [28, 70], the evening type has been proven to be a protective factor for suicide attempts. This suggests that disrupted sleep-wake cycles and misaligned circadian rhythms are more likely to cause suicide attempts than stable timing for rest and activity. Although, the evening type is associated with negative outcomes such as poor sleep quality, nightmares, and low mood. The moderately active type, which is characterized by persistent low alertness, has a lower impulsivity than the chaotic type among emerging adults with severe depressive symptoms [72]. Earlier research has shown that impulsivity is associated with unplanned responses that fail to consider their negative effects, leading to suicide attempts [29]. In agreement with earlier research on impulsivity, emerging adults with moderately chronotype were less likely to attempt suicide than emerging adults with the chaotic type. In addition, compared with the chaotic type, the daytime type was a protective factor for suicide attempts. Daytime type, associated with less daytime sleepiness, leads to increased serotonin concentration and activity, as well as increased HPA axis activity, which can prevent suicide attempts [73]. On the contrary, the chaotic type represents unstable sleep time and poor sleep quality, which is linked to mood disorders and increased impulsivity, thus making emerging adults more likely to attempt suicide [74, 75]. Our study results suggest that the chaotic chronotype is more accurate in predicting suicide attempts than the other chronotypes. Furthermore, there was no significant correlation between childhood trauma class and chronotype. This indicated that the impact of childhood trauma class and chronotype on suicide attempts is independent among emerging adults with severe depressive symptoms. Moreover, this study found that among emerging adults, being female and having an urban residence may result in a higher risk of suicide attempts, which is similar to a previous study among emerging adults [11]. The positive association between urban residence for emerging adults and suicide attempts also supported the results of earlier findings [13]. Thus, appropriate suicide attempt prevention programs and interventions should be targeted at female and urban-residing emerging adults.

The findings in the present study were subject to four limitations. First, as a cross-sectional design, this study cannot assume and establish the causality of childhood trauma class and chronotype to suicide attempts. Second, while the optimal model was selected according to the model fitting parameters of LCA, the class formed had no clinical significance. Moreover, as a large-scale study, this study only used the PHQ-9 scale to identify emerging adults with severe depressive symptoms rather than using a confirmed diagnosis such as the Structured Clinical Interview for DSM-V (SCID-V), which may lead to self-report bias. In addition, due to limited resources, we were unable to carry out any interventions for emerging adults with severe depressive symptoms. However, information about hospital help channels were provided to them. Finally, due to the influence of age on suicide attempts [12], the results of this study, based on the special population of emerging adults with severe depressive symptoms, should be carefully extended to other age groups.

## Conclusion

In conclusion, in order to better screen for the high risk of attempted suicide among emerging adults with severe depressive symptoms, a person-centered perspective was adopted to classify childhood trauma class into three classes. Specifically, the High-risk for childhood abuse class, where sexual, emotional, and physical abuse occurred at the same time, was a risk factor for suicide attempts. Furthermore, the evening, moderately active, and daytime chronotypes were also negatively associated with suicide attempts, when compared with the chaotic type. Applying our findings is important when targeting high-risk populations for death by suicide and can aid in developing appropriate interventions to reduce the risk of suicide attempts in emerging adults with severe depressive symptoms.

## Data Availability

The datasets used during the current study are available from the corresponding author on reasonable request.
